# The effects and the mechanisms of autophagy on the cancer-associated fibroblasts in cancer

**DOI:** 10.1186/s13046-019-1172-5

**Published:** 2019-04-23

**Authors:** Yuanliang Yan, Xi Chen, Xiang Wang, Zijin Zhao, Wenfeng Hu, Shuangshuang Zeng, Jie Wei, Xue Yang, Long Qian, Shuyi Zhou, Lunquan Sun, Zhicheng Gong, Zhijie Xu

**Affiliations:** 1 Department of Pharmacy, Xiangya Hospital, Central South University, 87 Xiangya Road, Changsha, 410008 Hunan China; 2 National Clinical Research Center for Geriatric Disorders, Xiangya Hospital, Central South University, 87 Xiangya Road, Changsha, 410008 Hunan China; 3 Department of Neurosurgery, Xiangya Hospital, Central South University, Changsha, 410008 Hunan China; 4 Center for Molecular Medicine, Xiangya Hospital, Central South University, Changsha, 410008 Hunan China; 5 Hunan Provincial People’s Hospital Xingsha Branch (People’s Hospital of Changsha County), Changsha, 410008 Hunan China; 6 Department of Pathology, Xiangya Hospital, Central South University, 87 Xiangya Road, Changsha, 410008 Hunan China

**Keywords:** Autophagy, Cancer associated fibroblasts, Cancer treatment

## Abstract

Cancer-associated fibroblasts (CAFs) plays an essential role in cancer cell growth, metabolism and immunoreaction. Autophagy is an intracellular self-degradative process that balances cell energy source and regulates tissue homeostasis. Targeting autophagy has gained interest with multiple preclinical and clinical trials, such as the pharmacological inhibitor chloroquine or the inducer rapamycin, especially in exploiting its ability to modulate the secretory capability of CAFs to enhance drug delivery or inhibit it to prevent its influence on cancer cell chemoresistance. In this review, we summarize the reports on autophagy in cancer-associated fibroblasts by detailing the mechanism and role of autophagy in CAFs, including the hypoxic-autophagy positive feedback cycle, the metabolic cross-talk between CAFs and tumors induced by autophagy, CAFs secreted cytokines promote cancer survival by secretory autophagy, CAFs autophagy-induced EMT, stemness, senescence and treatment sensitivity, as well as the research of antitumor chemicals, miRNAs and lncRNAs. Additionally, we discuss the evidence of molecules in CAFs that are relevant to autophagy and the contribution to sensitive treatments as a potential target for cancer treatment.

## Background

Since Yoshinori Ohsumi, the Nobelist in Physiology or Medicine, received his prize for elucidating the mechanisms of autophagy, more advances and highlights in the understanding of autophagy have been noted and shown to improve clinical outcomes in multiple areas, such as cancer [1], cardiovascular disease [2], obesity [3] and synapses [4]. Recent available data indicate that autophagy is a highly dynamic, multistep process that can be modulated at several steps, both positively and negatively. One critical point is that autophagy is commonly a conserved process in eukaryotes, involving the catabolism of multiple cytoplasmic components to maintain energy homeostasis and to protect cells against stress. Moreover, a critical process, known as selective autophagy, was more recently shown to selectively eliminate unwanted, potentially harmful cytosolic material, for example, damaged mitochondria or protein aggregates, thereby acting as a major cytoprotective system [5].

**Fig. 1:**
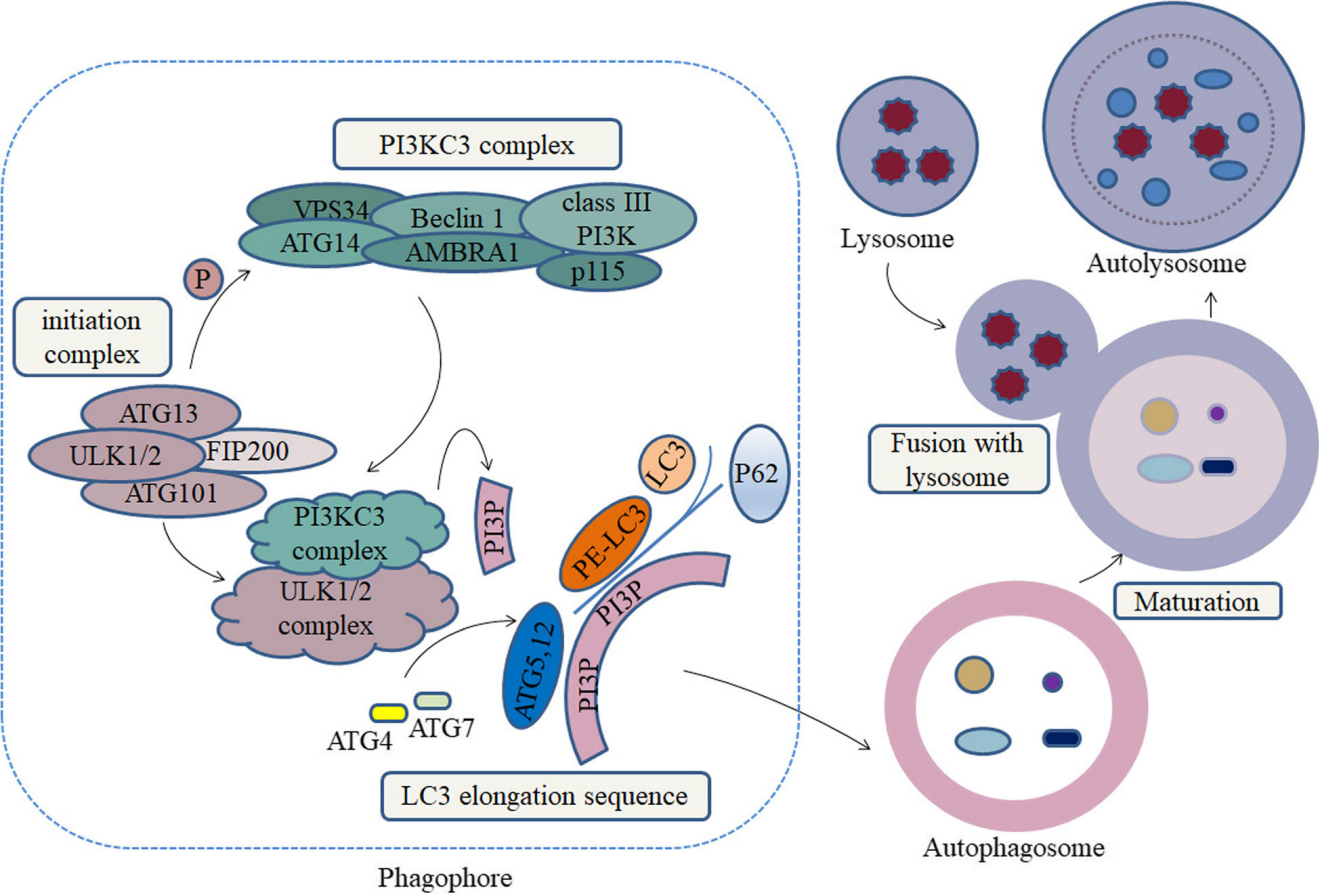
Autophagy-related proteins in the process of cell autophagy. The initial steps in autophagy include the nucleation, elongation, and maturation of an isolated membrane, usually called a phagophore. The formed phagophore then unites to form the autophagosome, and the fusion with a lysosome follows to form an autolysosome, where the captured materials and eliminated. Molecules which generally act as the markers of autophagy in the researches of CAFs and tumor, including LC3, ATGs, BECN1 and p62 were participated in the process of forming phagophore, autophagosome and autolysosome

Recently, studies found that the expression of autophagy-related proteins (ATGs) at CAFs or cancer cells, such as microtubule-associated protein light chain 3 (MAP1LC3/LC3), Beclin-1 (BECN1) and sequestosome 1 (SQSTM1 /p62) et al., might be promising indicators of for tumor recurrence and prognosis [6, 7]. During autophagy, cells recycle whole organelles and macro-molecules by three stages: 1) forming a phagophore by the initiation complex, including Unc-51 like autophagy activating kinase 1 (ULK1) complex and phosphatidylinositol 3-kinase catalytic subunit type 3 (PIK3C3)/vacuolar protein sorting protein 34 (Vps34) complex; 2) forming a compartment called autophagosome by two ubiquitin-like conjugating systems, Atg12-Atg5 and LC3 complex; 3) the maturation of autolysosome through the fusion between autophagosome and lysosomes (Fig. 1) [8, 9]. Phosphorylation of BECN1 and Vps34 triggers the activation of the PIK3C3 complex, resulting in local phosphatidylinositol-3-phosphate (PI3P) production [10]. Expansion of nascent precursor vesicles relies on the autophagosome protein LC3. Critical for this process is the phosphatidylethanolamine (PE) conjugated LC3-I and form of LC3-II [11]. In autophagy, p62 stably binds to the LC3-II protein acting a role as proteins trafficking in the context of assembling autophagosomes [12].

The tumor microenvironment has recently gained much attention as a critical determinant of tumor heterogeneity, initiation, progression, metastasis, and resistance to systemic therapies. The tumor microenvironment consists of immune cells (lymphocytes, natural killer cells, and antigen-presenting cells), stromal cells (including myofibroblasts), vasculature endothelial cells and adipocytes. In particular, cancer-associated fibroblasts (CAFs) are myofibroblast-like cells that induce the formation of a desmoplastic “reactive stroma”, compared with normal fibroblasts (FIBs), and promote tumor growth and aggressiveness [13]. The origin of CAFs is yet not well defined, but it is suggested that they arise from progenitors, in general mesenchymal stem cells (MSCs) or from other differentiated cells, such as smooth muscle cells (SMCs), cells of epithelial origin, endothelial cells, perivascular cells, and adipose tissue-derived stem cells [14]. Based on experimental evidence, CAFs markers, either CAF specific or CAF derived, have demonstrated an independent association with survival. This includes members of the platelet-derived growth factor receptor (PDGFR) family, transforming growth factor beta 1 (TGFB1) signaling, CAF-markers, such as podoplanin and fibroblast activation protein (FAP), as well as transcription factors (FoxF1) and secreted factors (matrix metalloproteinases (MMPs)) [15–17]. To identify specific markers to CAF subsets, Shicheng S et al. recently found two cell-surface molecules, CD10 and GPR77, which define a specific CAF subset that sustains cancer stemness and promotes tumor formation and chemoresistance [18].

**Fig. 2:**
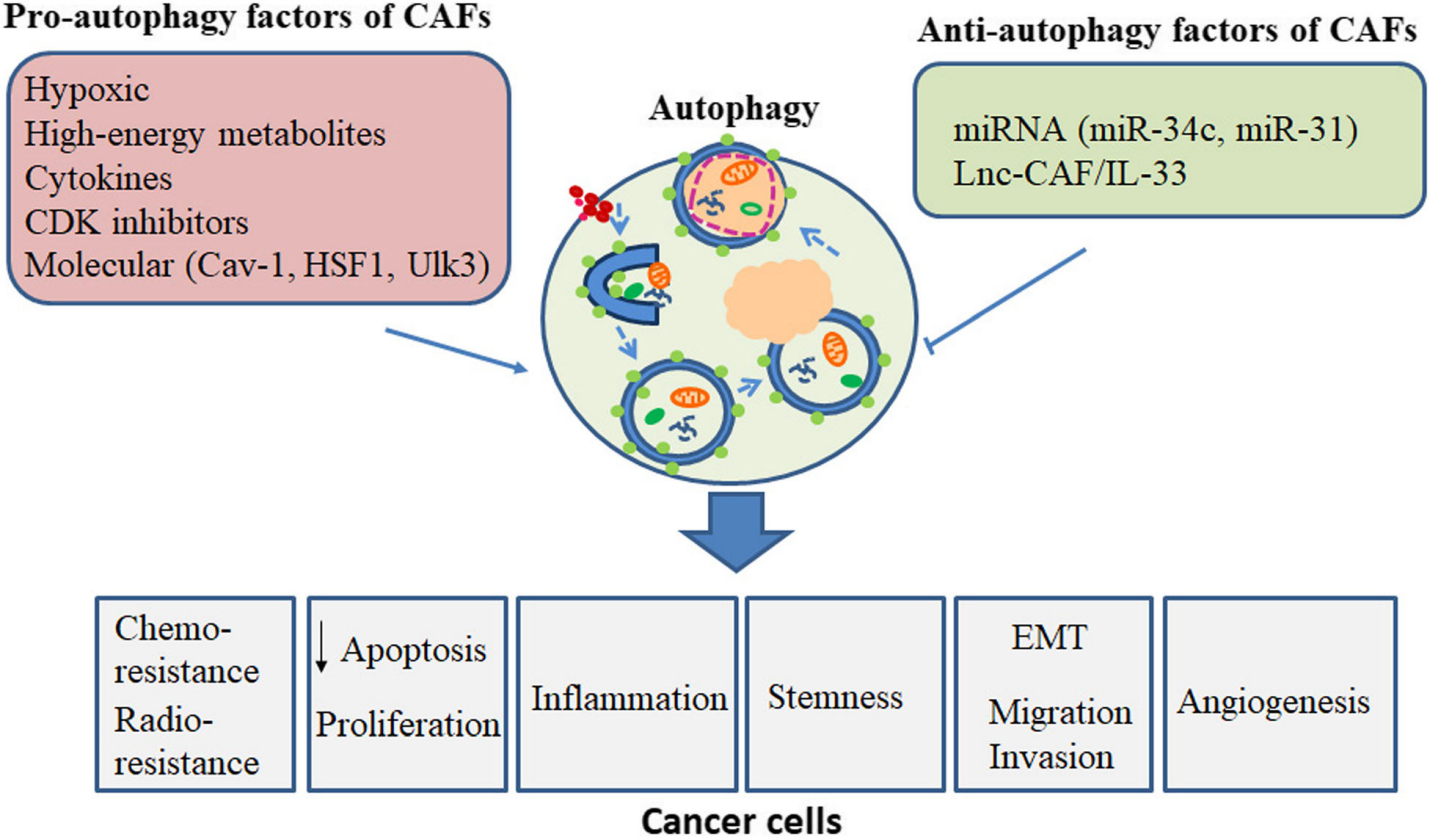
Overview of the autophagy-related process in CAFs. The function of autophagy in cancer-associated fibroblasts is mediated by the hypoxia pathway, glycolysis, senescence, antitumor chemicals, miRNAs and lncRNA, which then regulates tumor stemness, progression, resistance and the EMT process et al., leading to tumor progression and recurrence

The inconsistency of the CAF markers/gene signatures, which represent their presumably heterogeneous origin and function, suggests that the tumor and its microenvironment exhibit a considerable degree of plasticity in the paracancer and provides an alternative source for ‘active’ CAFs. For example, erlotinib-resistant cholangiocarcinoma cells display metastasis-associated signatures that correlate with a marked change in cell plasticity associated with epithelial-mesenchymal transition (EMT). In vivo, insulin receptor (IR)/ insulin-like growth factor 1 receptor (IGF1R) signaling positively regulates fibroblast proliferation and activation, reducing tumor growth [19]. Autophagy is believed to be one of the hallmarks of tumor cells, in parallel with genomic instability, provoking chronic inflammation, escape from the immune system, etc. [20, 21]. In this review, we focus on the function of autophagy in cancer-associated fibroblasts as two parts. In detail, we introduce the mechanism and role of autophagy in CAFs, including the hypoxic-autophagy positive feedback cycle; the metabolic cross-talk between CAFs and tumors induced by autophagy; CAF autophagy-induced tumor EMT; autophagic CAF-induced stemness in cancer; cyclin-dependent kinases (CDK) inhibitor-induced autophagy in CAFs; micro-RNA (miRNA) and long noncoding RNA (lncRNA) associated with autophagy in CAFs; and autophagy regulation in CAFs, mediating treatment sensitivity and the potential antitumor chemicals analyzed in CAFs (Fig. 2). In the second part, we critically discuss the evidence of molecules in CAFs that are relevant to autophagy from several studies to give future perspectives.

### The role and mechanism of autophagy in CAFs

Autophagy is a cellular catabolic mechanism that is responsible for the recycling of organelles, lipids and proteins, thereby helping to maintain cellular homeostasis and provide substrates for energy production. Previously, a study showed that CAFs undergo metabolic stress, which activates autophagy, for example, by inhibiting the AMPK-independent mTORC1 signaling pathways, to meet the increased energy demands of neighboring cells in the tumor microenvironment [22]. Another study illustrated that the CAFs in autophagy, stimulated by tumor cells, cause alanine secretion, which actually outcompetes glucose- and glutamine-derived carbon, in turn providing fuel for the tricarboxylic acid (TCA) cycle and thus for nonessential amino acids and lipid biosynthesis in its low-glucose microenvironment [23]. Obviously, this description gives a selective view and may exclude unknown factors and mechanisms.

### The role of CAFs autophagy

The role of the autophagy in CAF biology is complex, and it is shown to play critical roles that differ depending on the chemical treatment and biological context [24].The tumor mass reaction, including fibrotic stromal, also impairs the vasculature, leading to a highly hypoxic environment, due to the inhibition of the Notch/Hedgehog pathway, and a nutrient-poor environment, which suggests a link to the resulting “angiogenic switch” independent hypovascularity and perfusion impairment for tumor progression [25]. CAFs of tumor microenvironment positively influenced the proliferation and metabolism of cancer cells, through oxidative stress induced autophagy pathway which were initially induced by neighboring tumor cells [26]. Secretory autophagy is involved in the export of a variety of cellular cargoes. This includes leader less cytosolic proteins and inflammatory mediators, such as interleukin 1β (IL-1β), IL-6, IL-8 and IL-18 [27]. New J et al. showed that mitigating autophagy significantly reduced CAF-induced progression through IL-6, IL-8 and bFGF in neck squamous cell carcinoma. Treatment with autophagy target Vps34 inhibitor, SAR405, attenuated xenograft growth and inhibited the effects of standard therapy [28]. Similar results were found both in vitro (co-cultured model) or in vivo (xenografted model and clinical tissue) in cancer researches such as breast cancer, ovarian cancer, liver cancer, colorectal cancer and pancreatic adenocarcinoma [29, 30]. Additionally, there is extensive evidence in literature demonstrating that both radiation and chemotherapeutic drugs promote cytoprotective autophagy in tumor cells. Stress-related inducers triggered CAFs autophagy participate actively in tumor growth, invasiveness, and resistance to chemotherapy [31–33].

### The mechanism of CAFs autophagy

#### Hypoxic-autophagy positive feedback cycle in CAFs

Hypoxia is not only a prominent stressor in the microenvironment but also a crucial contributor to the heterogeneity of tumors to drive adaptations to support tumor growth and resistance to systemic therapies [34]. To understand the contribution of the microenvironment in promoting tumor growth and metabolic mechanisms, two Nobel Laureates formulated a hypothesis to explain the “fundamental basis” of cancer. In the “Warburg Effect” hypothesis, cancer cells produce energy via the conversion of glucose into lactate, despite the presence of oxygen, a process known as aerobic glycolysis. The “Pasteur effect” further described the inhibiting effect of glycolysis upon oxygen, confirming aerobic glycolysis as a hallmark of the cancer phenotype. This glycolytic pathway is accentuated under hypoxia, which acts pleiotropically to upregulate glucose transporters and multiple enzymes by independently increasing the levels of the HIF1a and HIF2a transcription factors [35]. Lisanti MP et al. proposed the “tumor-stroma co-evolution” model, indicating that breast cancer cells induced oxidative stress in adjacent stromal fibroblasts and upregulated autophagy and mitophagy in the tumor microenvironment [36]. This reliance on the autophagy and oxidative stress pathways demonstrated in CAFs is consistent within many tumors.

**Fig. 3:**
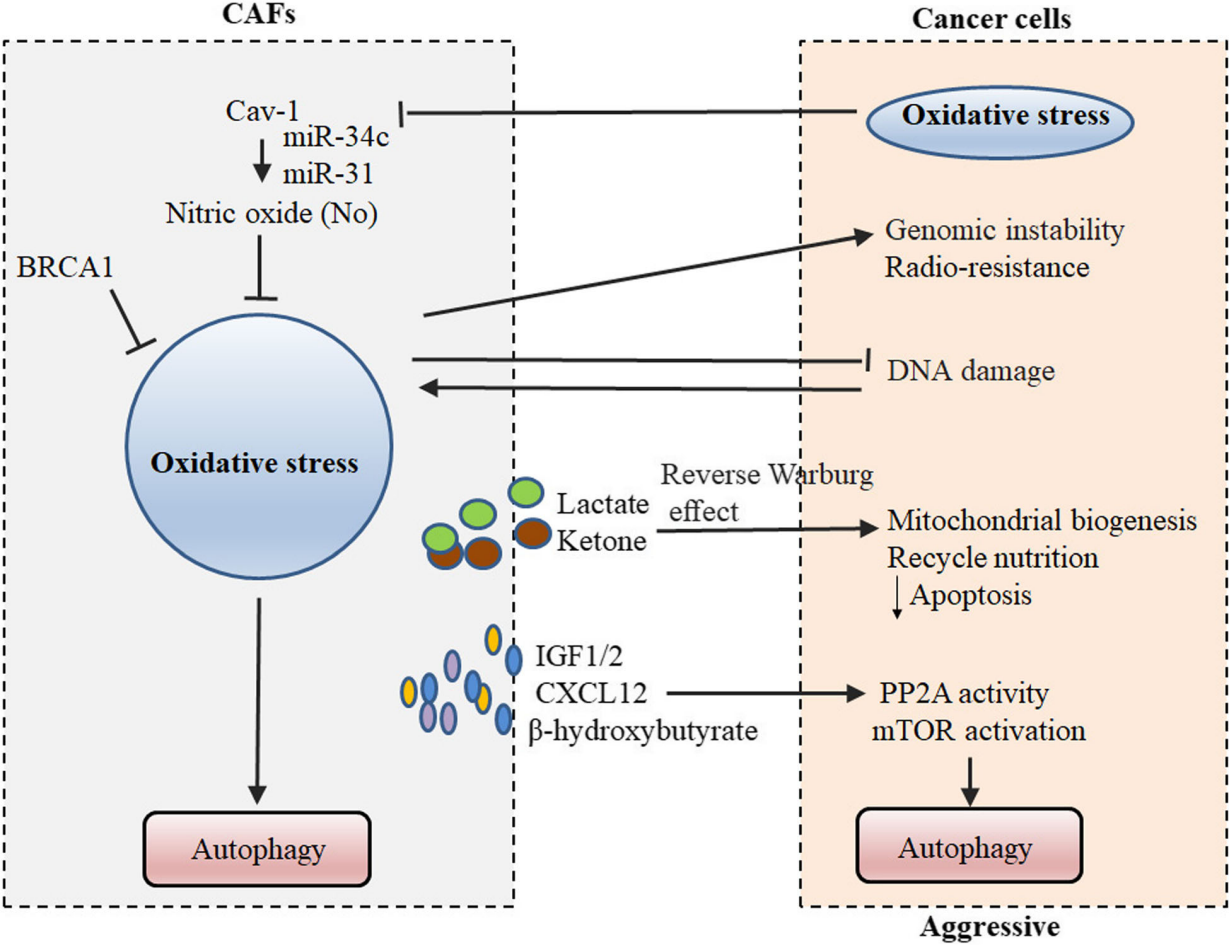
Hypoxic-autophagy positive feedback cycle in CAFs. In a coculture system of different cancers, tumor cells induce oxidative stress in adjacent stromal CAFs and upregulate autophagy and mitophagy in the tumor microenvironment

By the overwhelming intercellular change of oxidative stress, such a positive-feedback cycle turns a condition of autophagy in CAFs or cancer cells (Fig. 3). Data on the stroma-tumor crosstalk indicated that caveolin-1 (CAV1) and breast cancer type 1 susceptibility protein (BRCA1) involved in oxidative stress pathway in CAFs. The membrane protein CAV1 possesses tumor-suppressor properties within stromal cells, whereas downregulation of stromal CAV1 correlated with cancer progression, invasion and metastasis and thus, a worse clinical outcome [37]. A coculture system, by which a loss of stromal fibroblast CAV1 induces a “lethal tumor microenvironment,” demonstrated that MCF7 epithelial cancer cells induce oxidative stress in adjacent CAFs, resulting in the autophagic/lysosomal degradation of stromal CAV1 [38, 39]. Tumor cell-derived reactive oxygen species (ROS) decrease the expression of CAV1 in CAFs [40]. Consistent with the metabolic findings, certain miRs are found to be associated with oxidative stress (miR-34c) or activation of the hypoxic response/HIF1a (miR-31), which is sufficient to drive autophagy/mitophagy. Upregulating oxidative stress in CAFs is sufficient to induce genomic instability in adjacent cancer cells, via a bystander effect, potentially increasing their aggressive behavior [41]. Studies demonstrated that BRCA1 is mutated in 45% of hereditary breast cancers, which has been described recently as an autophagy inhibitor [42, 43] Salem AF et al. reported that BRCA1 induces several antioxidant genes that are responsible for ROS inhibition, and knockdown of BRCA1 in CAFs is able to significantly promote tumor growth [44]. Preclinical studies using xenografts demonstrate that shBRCA1 fibroblasts, with an increase in markers of autophagy and mitophagy, induced an ~ 2.2-fold increase in tumor growth when coinjected with MDA-MB-231 cells into nude mice via ketone production [45].

Additionally, oxidative metabolism of CAFs provide nutrients (such as ketone) and cytokines to stimulate mitochondrial biogenesis and autophagy, by a reverse Warburg effect, in adjacent cancer cells. Cancer cells escape oxidative mitochondrial damage and apoptosis by the upregulation of antioxidant enzymes, such as peroxiredoxin-1 [36]. In a noncontact coculture system of human colorectal/ ovarian fibroblasts and cancer cells, CAFs positively influence the metabolism of cancer cells, through the autophagy and oxidative stress pathways, which are initially induced by neighboring tumor cells [26, 46]. Further research indicates that CAFs promote irradiated cancer cell recovery and tumor regrowth postradiation. In in vitro and in vivo xenograft models of lung cancer and melanoma cells, CAFs produce IGF1/2, CXCL12 and β-hydroxybutyrate and increase the level of ROS postradiation, which enhances protein phosphatase 2A (PP2A) activity, resulting in repressing mTOR activation and increasing autophagy in cancer cells postradiation. A further point mutation result suggests that the oxidation of PP2Ac Cys251 could enhance PP2A activity, validating the IGF2 function through above-mentioned mechanism [32]. This postradiation result indicates that CAFs play key roles in irradiating cancer cell recovery, which is in accordance with the previous observations that preexisting CAFs enhance the radiation resistance of tumor cells [47]. Although the role of hypoxic stress in the crosstalk among CAFs and tumor cells is not fully elucidated, it is widely appreciated that the hypoxic zone in CAFs induces autophagy in themselves or by a paracrine pathway of secreting cytokines in tumor cells.

#### Metabolic cross-talk between CAFs and the tumor induced by autophagy

**Fig. 4:**
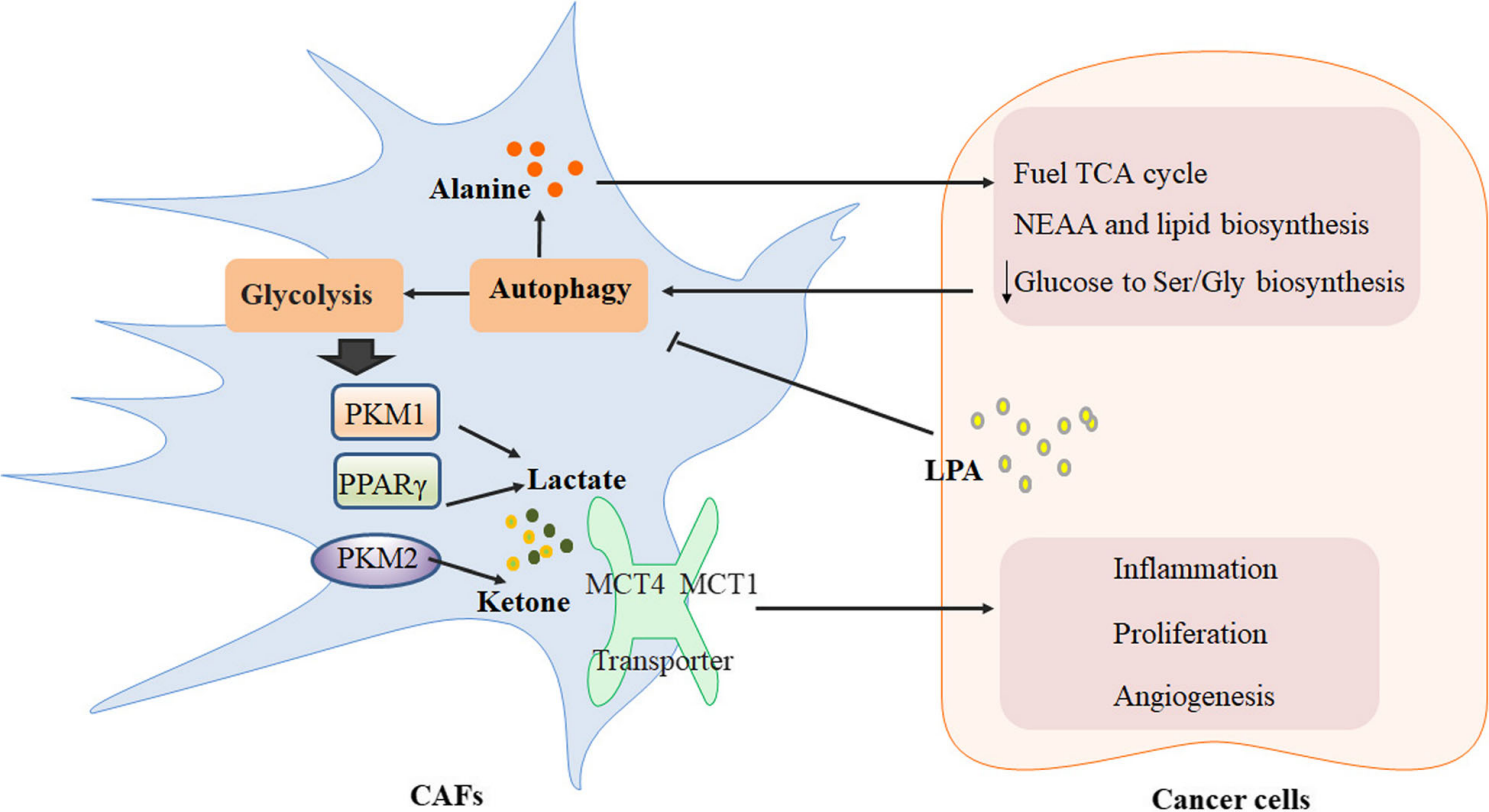
Metabolic cross-talk between CAFs and tumors induced by autophagy. Via the secretion of high-energy metabolites by the tumor stroma, such as lactate, ketone and alanine, aerobic glycolysis and/or autophagy is enhanced in the CAFs to support cancer cell growth and an aggressive behavior

With the novel concepts of the “reverse Warburg effect” and the “autophagic tumor stroma model of cancer metabolism” that Michael P et al. proposed, an in vitro study demonstrates that the enhanced aerobic glycolysis and/or autophagy in the CAFs supports epithelial cancer cell growth and aggressive behavior via the secretion of high-energy metabolites by the tumor stroma [38, 48] (Fig. 4). These nutrients include ketones and lactate, as well as chemical building blocks such as amino acids (glutamine) and nucleotides. Lactate and ketones serve as fuel for cancer cell oxidative metabolism, and building blocks sustain the anabolic needs of the rapidly proliferating cancer cells. A further in vivo study of a xenograft model shows that the recombinant overexpression of pyruvate kinase M (PKM1 and PKM2), a key enzyme in the glycolytic pathway, is sufficient to promote the growth of breast cancer cells in human fibroblasts, increasing tumor mass and tumor volume, without an increase in tumor angiogenesis [49]. The expression of PKM1 enhances the glycolytic power of stromal cells, with an increased output of lactate, and induces tumor inflammation. PKM2 increases the output of the ketone body 3-hydroxybutyrate, triggering a “pseudo-starvation” response and the induction of an NFκB-dependent autophagic program in stromal cells. A similar result was found in Paola Avena’s study. CAFs with activated peroxisome proliferator-activated receptor γ (PPARγ), display metabolic features with increased autophagy, glycolysis and senescence [50]. Overexpressing PPARγ in the tumor stroma reveals a 70% increase in L-lactate accumulation, relative to control fibroblasts. The controversial role of PPARγ, showing either an autophagy-induced protumorigenic effect in the CAFs or antineoplastic effects in epithelial cancer cells, suggests that the activation of an autophagic program has both pro- or antitumorigenic effects depending on the cell compartment in which it occurs [51, 52]. Recently, a small signaling phospholipid suffices to activate mTORC1 and suppress autophagy [53], Lysophosphatidic acid (LPA), was identified a role for LPA-HIF1α signaling-hub in the maintenance of the glycolytic-phenotype in CAFs [54]. The signaling locus for CAF-phenotype targeted inhibition of LPA-mediated metabolic reprogramming in CAFs may represent an adjuvant therapy in ovarian cancer. Pancreatic stellate cells (PSCs) are the precursors of CAFs, which potentiate pancreatic tumor growth and progression [55]. A previous study illustrates that an intratumoral metabolic cross-talk occurs between different populations of cells in a tumor [56]. Recently, the alterations in lactate and alanine were reported using imaging studies during pancreatic cancer progression in mouse models [57]. Consistent with the previous studies, Cristovão M et al. showed that PSCs are critical for pancreatic ductal adenocarcinoma (PDAC) metabolism through the secretion of nonessential amino acids (NEAA). Autophagic alanine secretion outcompetes glucose and glutamine-derived carbon in PDAC to fuel the tricarboxylic acid (TCA) cycle, and thus, NEAA and lipid biosynthesis, decreasing the tumor’s dependence on glucose and serum-derived nutrients in microenvironment. Within this shift in fuel source, the alanine secretion by PSCs is dependent on PSC autophagy, a process that is stimulated by cancer cells [23]. This finding indicates a novel metabolic interaction of the tumor stroma, in which alanine acts as an alternative carbon source, an effect that is not even recapitulated with exogenous lactate.

In addition, the metabolic alterations in lung cancer-associated fibroblasts were determined by mass spectrometry-based profiling of the abundances of 203 biochemicals of 46 metabolic pathways/groups to compare primary human lung tumor CAFs to “normal” fibroblasts (NFs) [58]. Although the results showed no differences in the individual metabolites distinguishing CAFs from NFs, significant differences were found between CAFs and NFs in the steady-state abundances of the metabolites of select metabolic pathways. The distinct roles of CAFs are related to the tumor’s glycolytic capacity, which is reflected by the metabolic differences between the CAFs from high and low glycolytic tumors. Dipeptide levels are significantly increased in CAFs, which is a general characteristic of CAFs that is reported to be correlated with the glycolytic activity of the tumor [59]. Most interestingly, an increase in basal macroautophagy was found, which might account for the increase in dipeptide levels. The difference between CAFs and NFs is demonstrated in the induction of autophagy promoted by reduced glucose, which, taken together, suggests that the increased autophagy may account for metabolic differences between CAFs and NFs.

#### CAFs secreted cytokines promote cancer survival by secretory autophagy

The tumor microenvironment is a specialized niche that impacts malignant cells directly and indirectly through stromal cells that support tumor growth [60]. Several cytokines were identified, including IL-6, IL-8, IGF1, IGF2, and CXCL12, all of which promoted survival of cancer cells [30, 61]. Autophagy is closely intertwined with inflammatory and immune responses. Proinflammatory cytokines such as IFN-γ, TNF-α, IL-17, and cytokines of the IL-1 family, regulate or be regulated through autophagy mediated this interaction [62, 63]. A concept discovered less than half a decade ago, secretory autophagy, refer to CAFs secrete soluble factors through autophagy, has a multifaceted impact on the cancer microenvironment [64]. The mechanisms of CAF-tumor cell interaction have been showed including paracrine signaling and exosomal transfer mediated by cytokines such as IL-6 and GM-CSF [65]. Lu H et al. reported that IL-6 could induce autophagy by expressing NS5ATP9, while NS5ATP9 upregulated IL-6 levels in turn, which further induced autophagy [66]. TGFβ1 small latent complex could select by golgi reassembly stacking protein 2 and secret via MAP1LC3/LC3-positive secretory autophagosomes through an unconventional pathway in fibroblasts and macrophages [67].

Recently, Thongchot S et al. firstly demonstrated that CAFs secretory products directly affect the regulation of autophagy and consequently the behavior of cholangiocarcinoma cells. Specifically, resveratrol has the potential to abrogate the effects of IL-6 mediated motility by CAFs and reverted the N-to E-cadherin switch in migrating cells [68]. Ferraresi A et al. showed that polyphenol resveratrol can oppose the stimulatory effect of IL-6 on cell migration through epigenetic up-regulation of autophagy of ovarian cancer cells [69]. Proteomic studies demonstrated that bortezomib could triggers CAFs to produce high levels of IL-6, IL-8, IGF-1, and TGFβ, then activate oxidative stress and pro-survival autophagy in multiple myeloma [70]. Li WL et al. found that IL-13 regulates BECN1 and LC3B expression through IKKβ/NFκBp65 in fbroblasts cocultured with breast cancer cells [71]. Stromal cyclin D1 increased in fibroblasts can promotes heterotypic immune signaling by increased secretion of proinflammatory cytokines (CCL2, CCL7, CCL11, CXCL1, CXCL5, CXCL9, CXCL12), CSF (CSF1, GM-CSF1) and osteopontin (OPN) [72].

Moreover, blocking autophagy in CAFs could supports chemotherapy through proliferation inhibition in pancreatic, oral squamous cell carcinoma and pancreatic adenocarcinoma cancer cells [73–75]. Radiation-induced rescue effect is closely related to radiation-induced bystander effect and describes the phenomenon that irradiated cells derive benefits from feedback signals released from bystander unirradiated cells, then alleviate the harmful radiobiological effects. A study found that bystander factors released from irradiated cells could induced autophagy and activated STAT3 to produce IL-6 in bystander unirradiated cells, which activated NF-κB pathway in irradiated cells [76]. These data indicated that treatment with targeting epigenetic changes of CAFs mediated autophagy that contrast the malignant phenotype could act as potential adjuvant chemotherapeutics in chemoradiotherapy of tumor.

#### CAFs autophagy-induced tumor EMT

CAFs autophagy can affect the metastatic behavior of cancer cells by inducing epithelial-to-mesenchymal transition. Previous study found that CAFs could induce EMT in MCF7 breast cancer and MCF10A breast epithelial cell lines, indicating that CAFs contribute to malignant phenotype and doxorubicin sensibility in breast cancer [77]. Recently, Wang M et al. showed CAFs autophagy induced triple-negative breast cancer (TNBC) cells to engage in the EMT process through the Wnt/β-catenin pathway, to enhance TNBC cell migration, invasion, and proliferation [29]. In a coculture model of TNBC, autophagy-relevant BECN1 and LC3-II/I protein conversion levels in the CAFs are higher than those in NFs. The significant levels of downregulated E-cadherin and upregulated vimentin/N-cadherin are found in the TNBC cells from the CAF group. This effect was reversed when CAFs were previously cultured with autophagy inhibitor 3-MA. Epidermal growth factor receptor (EGFR) plays an integral role in the tumorigenic process, which makes it an attractive target for pharmacologic inhibition by the induction of autophagic cancer cell death [78, 79]. Using an adenocarcinoma model of epidermal growth factor receptor tyrosine kinase inhibitors (EGFR-TKI)–acquired resistance, an EMT subpopulation of CAFs was isolated and was found to be tumorigenic and expressed the biomarker of gefitinib resistance, epithelial membrane protein-1. The evidence suggests that paracrine factors secreted from the EGFR-TKI–resistant CAFs mitigate the EGFR-TKI–mediated blockade of pEGFR and pMAPK in cocultured tumor cells, regardless of their EGFR mutational status [80]. This result demonstrates that the tumor stroma may, through autophagy, modify the acquisition of EGFR-TKI resistance and further contribute to promoting drug resistance.

#### Autophagic CAFs induce stemness in cancer

Cancer stem cells (CSCs) and their microenvironmental niche are involved in tumor maintenance and recurrence due to their ability to survive traditional therapies [81]. CAFs are the predominant component of the cancer microenvironment, and they play a role in the occurrence and progression of malignant tumors, such as luminal breast cancer [82]. It is reported that CAFs from breast cancer have autophagic activity, involving the malignant potential and chemoresistance of the tumor cells [83]. Notably, recent work declaims that high-mobility group box 1 (HMGB1) cytokines secreted by CAFs in the niche through an autophagy-based unconventional secretion are involved in the cross-talk between CSCs and CAFs to promote the tumorigenesis and self-renewal of CSCs [6]. Mechanistically, HMGB1 activates its receptor, Toll-like receptor (TLR) 4, to enhance the stemness and tumorigenicity of luminal breast cancer cells. Furthermore, immunohistochemistry results of luminal breast cancer specimens are consistent with this founding, suggesting that a high autophagy level predicts an increased relapse rate and a poorer prognosis, as the potential therapeutic targets.

#### CDK inhibitors induce autophagy in CAFs

Recent studies show that senescence and autophagy may be part of the same metabolic program, known as the autophagy-senescence transition (AST) [84]. The increased expression of autophagy in stromal fibroblasts is sufficient to induce the onset of constitutive autophagy as well as the development of senescence [85, 86]. Capparelli C et al. showed that the recombinant expression of CDK inhibitors (p16/p19/p21) is sufficient to induce autophagy, driving the senescence-autophagy transition (SAT) in CAFs [87]. Thus, both SAT and AST result in mitochondrial dysfunction and a metabolic shift toward glycolysis, “powering down” cells during cell cycle arrest. In conclusion, cell cycle arrest, autophagy and senescence are all part of the same metabolic program that occurs in response to cellular stress, providing a new genetically tractable model for understanding the metabolic role of “host aging” in promoting tumor growth and metastasis by providing a “fertile” local microenvironment.

#### miRNAs and lncRNAs associated with autophagy in CAFs

Studies have indicated that miRNAs and lncRNAs regulate the cell growth, apoptosis, and metastasis of cancer cells [88]. A growing number of studies confirm that miRNAs or lncRNAs play essential roles as biomarkers in the diagnosis of cancers and as target molecules for cancer treatment [89, 90]. A previous study proposed that the levels of miR-31 could be assayed using the serum or plasma from cancer patients or could be assessed directly from excised tumor tissue as key biomarkers [41]. By coculturing CAFs and tumor cells, the authors confirmed that miR-31 significantly inhibited the autophagy of colorectal cancer CAFs at both the protein and mRNA levels and further affected the proliferation and radiosensitivity (mainly radiation-induced apoptosis) of colorectal cancer cells [91]. Metastasis-associated lung adenocarcinoma transcript 1 (MALAT1) was up-regulated lncRNA in many tumors and associated with cancer cell metastasis and recurrence. Hu J et al. found that E3 ubiquitin ligase MARCH7 could interaction with MALAT1, regulating TGFβR2-Smad2/3-MALAT1/MARCH7/ATG7 feedback loop, and mediated autophagy, migration and invasion in ovarian cancer [92]. A further study identified a stromal lncRNA signature during the transformation of CAFs from NFs in oral squamous cell carcinoma (OSCC) using RNA sequencing. An uncharacterized RNA, FLJ22447, which was remarkably upregulated in CAFs, referred to as Lnc-CAF, upregulated IL-33 levels and prevented the p62-dependent autophagy-lysosome degradation of IL-33, which was independent of the lncRNA-protein scaffold effects. After Lnc-CAF knockdown, the interaction between p62 and IL-33 increased, leading to the degradation of IL-33 via the upregulation of selective autophagy. An in vivo study also demonstrated a stromal Lnc-CAF signature as an oncogene, promoting OSCC [93]. Further studies on the interactions between miRNAs or lncRNAs in the tumor stromal compartment must be investigated.

#### Autophagy regulation in CAFs mediates treatment sensitivity

Autophagy and mitochondrial dynamics have recently been implicated in the radioresistance and chemoresistance of cancer cells, such as sunitinib, cisplatin, and erlotinib [34, 94–96]. In a recent study, enhanced basal autophagy in CAFs facilitated the secretion of tumor-promoting factors, notably IL6 and IL8, in neck squamous cell carcinoma (HNSCC). The secretion of IL6, IL8, and basic fibroblast growth factor (bFGF) is, at least in part, responsible for the promotion of CAF autophagy, which is further maintained through an IL6 and IL8 autocrine feedback. The amelioration of HNSCC autophagy by an autophagy inhibitor, such as chloroquine or SAR405, gives an indication to the potential therapeutic value of a combinatorial targeting of autophagy with standard-of-care therapy [28]. PCI-5002, a zinc ionophore, decreases cell proliferation in treated A549 lung cancer cells and PC3 prostate cancer cells [97]. In in vitro cancer models, decreased apoptosis in Bax/Bak^−/−^ mouse embryonic fibroblasts increases autophagy and is associated with the radiosensitization of cells compared with the wild-type (WT) mouse [98, 99]. Autophagic conditions are often found in the tumor stroma, where CSL/RBPJk levels are down-modulated. Goruppi S et al. identified a key role for autophagy in the degradation of CSL through a direct interaction with the p62 adaptor, regulating CAF activation and autophagy [31, 100]. Taken together, these data support the mediation of autophagy in the tumor stroma as an effective therapeutic approach for the prevention of local cancer recurrence.

#### Molecular in CAFs relevant to autophagy

Caveolin-1 (CAV1) is a known a biomarker of the catabolic CAF phenotype, which is reversible upon treatment with antioxidants and is a strong predictor of a poor clinical outcome in various types of human cancers [101]. In clinical research of gastric cancer (GC), as poor prognosis marker, the low expression of fibroblastic CAV1 is found with positive fibroblastic LC3B [102]. The transcription factor HSF1 indirectly promotes tumorigenesis in several types of cancer cells by enabling proliferation, invasion and metastasis [103]. Much research indicates that HSF1 upregulates ATG4B expression and enhances autophagy by epirubicin-induced protective or selective ways [104, 105]. It is reported that HSF1 is frequently activated in CAFs though central stromal signaling molecules, such as TGFβ and SDF1, where it is a potent enabler of malignancy and may be associated with the autophagy process [106]. The autophagy inducer, ULK3, in human fibroblasts, is critical for the convergent control of CAF activation by the CSL/RBP-Jκ protein and glioma-associated transcription factors [107]. All of these autophagy-relevant molecules involved in CAF conversion are attractive targets for stroma-focused anticancer intervention.

## Discussion and conclusion

Even though the concept of tumor stromal cross-talk is accepted [108, 109], Moinfar F et al. showed that genetic events, specifically the loss-of-heterozygosity (LOH) at microsatellite markers on 11q21-q23, 3p14.2, 16q23-q24, and 17q24, accumulate and contribute to tumorigenesis in breast cancer as stroma polymorphic microsatellite markers [110]. This observation suggests that somatic genetic alterations not only occur in the tumor stroma but also play an important role in the development and/or progression of solid tumors. Given the putative role of autophagy in CAFs, the identification of agents that differentially modulate the autophagy responses is important in developing a clinical armamentarium to modulate CAFs or their phenotypic expression. To date, three forms of autophagy are known-chaperone-mediated autophagy, microautophagy, and macroautophagy [111]. Importantly, investigators need to determine whether they are evaluating levels of early or late autophagic compartments, or autophagic flux, which is a form involved in CAF intervention. Many cancer types have a high stromal content, such as pancreatic cancer, non-small cell lung cancer (NSCLC), TNBC and sarcoma et al., contributing to low response rates to current therapies and a poor long-term survival [112, 113]. Emerging evidence suggests that the stromal compartment impedes the effective uptake of chemotherapeutics such as Letrozole and polygonatum, shaping the antitumor immunity and responsiveness to immunotherapy by autophagy [114–118]. All of these results indicate a disruption in the CAFs to improve drug efficiency, and this is a potential strategy that must be further pursued in the future both preclinically and in early clinical trials in stroma-rich tumors. Further original research and clinical trials are needed to make a significant impact for patients, with potential therapeutic strategies using a CAF-focused anticancer intervention. In development or currently underway, we believe that CAFs can help to answer the question of tumor heterogeneity and the inconsistent results of in vivo/in vitro models. Thus, CAFs could be a new model to minimize the gap between experiment and reality.

## Abbreviations

ATGs: Autophagy-related proteins
CAFs: Cancer-associated fibroblasts
CSCs: Cancer stem cells
EGFR: Epidermal growth factor receptor
EMT: Epithelial-mesenchymal transition
FAP: Fibroblast activation protein
HMGB1: High-mobility group box 1
IGF1R: Insulin-like growth factor 1 receptor
IL-1β: Interleukin 1β
IR: Insulin receptor
lncRNA: Long noncoding RNA
LPA: Lysophosphatidic acid
miRNA: micro-RNA
MMPs: Matrix metalloproteinases
NEAA: Nonessential amino acids
NSCLC: Non-small cell lung cancer
PDAC: Pancreatic ductal adenocarcinoma
PDGFR: Platelet-derived growth factor receptor
PE: Phosphatidylethanolamine
PI3P: Phosphatidylinositol-3-phosphate
PIK3C3: Phosphatidylinositol 3-kinase catalytic subunit type 3
PP2A: Protein phosphatase 2A
PPARγ: Peroxisome proliferator-activated receptor γ
PSCs: Pancreatic stellate cells
ROS: Reactive oxygen species
TCA: Tricarboxylic acid
TGFB1: Transforming growth factor beta 1
ULK1: Unc-51 like autophagy activating kinase 1
Vps34: Vacuolar protein sorting protein 34
